# Green Synthesis of Oat-Derived Carbon Quantum Dot/Gelatin Hydrogel Scaffolds: Enhanced Structural Stability and Bioactivity for Potential Bone Repair

**DOI:** 10.3390/gels12090757

**Published:** 2026-08-24

**Authors:** Aya Samy, Wessam Omara, Asmaa M. Abd El-Aziz, Azza El-Maghraby, Khaled O. Sebakhy, Sherif H. Kandil, Ahmed Abd El-Fattah

**Affiliations:** 1 Department of Materials Science, Institute of Graduate Studies and Research, Alexandria University, El-Shatby, Alexandria 21526, Egypt; lalaaya917@gmail.com (A.S.); wessam.omara@alexu.edu.eg (W.O.); s.kandil@usa.net (S.H.K.); 2 Fabrication Technology Research Department, Advanced Technology and New Materials Research Institute, City of Scientific Research and Technological Applications (SRTA-City), Borg Al-Arab, Alexandria 21934, Egypt; aabdelaziz@srtacity.sci.eg (A.M.A.E.-A.); maghrabyazza@yahoo.com (A.E.-M.); 3 Department of Chemical Engineering, School of Engineering (EDICT), Bahrain Polytechnic, Isa Town P.O. Box 33349, Bahrain; khaled.sebakhy@polytechnic.bh; 4 Department of Chemistry, College of Science, University of Bahrain, Sakhir P.O. Box 32038, Bahrain

**Keywords:** carbon quantum dots, oatmeal, gelatin, hydrogel composites, bioactivity, cytotoxicity

## Abstract

The development of sustainable, biocompatible scaffolds with enhanced structural stability remains a primary challenge in bone tissue engineering. In this study, structurally reinforced nanocomposite scaffolds were successfully fabricated by integrating green-synthesized carbon quantum dots (CQDs) into a gelatin (G) matrix, offering an innovative platform that mimics the organic–inorganic interfaces of natural bone tissue. The CQDs were derived from oatmeal via a sustainable, green hydrothermal route, serving simultaneously as zero-dimensional reinforcing fillers and bioactive agents within the biopolymer network. To ensure an additive-free fabrication process that avoids toxic chemical cross-linkers, dehydrothermal (DHT) treatment was employed, successfully modulating the interfacial and chemical cross-linking interactions between the gelatin chains and the oxygen-rich surface groups of the CQDs. Structural characterization confirmed the uniform dispersion of CQDs (average diameter 7–8 nm) within the porous gelatin framework. The incorporation of CQDs significantly improved the physicochemical properties of the scaffolds; the G/CQD 5% formulation emerged as the optimal composition, exhibiting a 118% increase in compression modulus compared to pristine gelatin. The composite demonstrated tuned swelling kinetics and a significantly reduced degradation rate, restricting mass loss after 14 days of incubation to approximately 24% compared to 40% for pristine gelatin, which is essential for maintaining a structural template during the initial stages of tissue formation. Bioactivity assays in simulated body fluid (SBF) confirmed the rapid, biomimetic induction of a crystalline hydroxyapatite layer with a natural Ca/P ratio of 1.61 within 14 days. Furthermore, preliminary in vitro assessments using Human Skin Fibroblasts (HSFs) confirmed excellent general cytocompatibility, with cell viability exceeding 90%. This study highlights the unique potential of utilizing biomass-derived carbon nanostructures and clean manufacturing processing to engineer multifunctional scaffolds with enhanced structural stability and intrinsic bioactivity for potential bone defect repairs.

## 1. Introduction

Bone tissue engineering (BTE) has emerged as a critical field for treating significant bone defects and fractures that exceed the body’s natural regenerative capacity. Central to BTE is the development of 3D scaffolds that provide a temporary microenvironment for cell attachment, proliferation, and subsequent biomineralization [1,2,3]. Among various biomaterials, hydrogels are widely utilized due to their high water content and structural similarity to the natural extracellular matrix (ECM) [4].

Gelatin (G), a collagen derivative obtained through the partial hydrolysis of collagen, is particularly favored for its excellent biocompatibility, low immunogenicity, and presence of functional amino acid residues that facilitate cell–material interactions [5]. However, the application of pristine gelatin in bone repair is often limited by poor mechanical toughness and rapid enzymatic degradation under physiological conditions [6,7].

To overcome these structural limitations, recent strategies have focused on the development of nanocomposite hydrogels, where functional nanomaterials are incorporated to reinforce the underlying polymer network [8,9]. Within this domain, carbon quantum dots (CQDs) have gained significant attention due to their ultra-small size (<10 nm), high photoluminescence, and abundant surface functional groups, such as carboxyl, hydroxyl, and carbonyl moieties. These surface functionalities allow CQDs to serve as multifunctional nanoscale cross-linking nodes by participating in dense hydrogen bonding and interfacial coupling with polymer matrices [10]. Unlike traditional metallic or semiconductor quantum dots, CQDs exhibit negligible cytotoxicity, superior cytocompatibility, and unique biological traits, such as intrinsic antioxidant activity and the ability to modulate cellular pathways [11].

Driven by these advantages, the paradigm of sustainable nanotechnology has accelerated interest in the green synthesis of CQDs using biomass waste or natural precursors. Utilizing renewable sources such as coconut husks, fruit extracts, or cereals offers a cost-effective and environmentally friendly alternative to traditional chemical precursors [10,12,13].

Despite these advances, the existing literature on biomass-derived CQD-containing hydrogel systems reveals critical gaps that limit their clinical potential. While various fruit, plant, and cereal-derived carbon dots have been integrated into biopolymer networks, many reported formulations suffer from a lack of systematic loading optimization [14]. Researchers often investigate a narrow concentration range that fails to determine the precise percolation threshold required to simultaneously balance mechanical reinforcement, swelling kinetics, and degradation profiles.

Furthermore, many current systems still rely on conventional, toxic chemical cross-linking agents (e.g., glutaraldehyde, EDC/NHS) to achieve structural stability, which compromises the green integrity of the biomaterial and introduces risks of residual chemical toxicity. Mechanistically, existing studies frequently restrict their bioactivity evaluations to superficial morphological observations of cell growth, leaving the systematic, quantitative kinetics of simulated body fluid (SBF) mineralization underexplored and insufficiently integrated with rigorous structural and elemental analyses [15].

To address these limitations, the strategic selection of the carbon precursor is paramount. In this study, oatmeal was selected as a sustainable, inexpensive, and highly renewable biomass precursor for hydrothermal CQD synthesis. It possesses a unique macromolecular blueprint rich in complex carbohydrates (starch) and proteins, alongside essential trace minerals. Its hydrothermal carbonization generates oxygen-functionalized nanodomains with a high nitrogen-to-carbon ratio, driving the in situ formation of self-doped functional groups [16]. These surface functionalities are critical for two reasons: they facilitate robust interfacial and chemical interactions with the amino acid backbones of gelatin, and they act as potent, negatively charged nucleation sites that accelerate calcium and phosphate ion deposition during biomineralization.

The core novelty of this work lies in the synergistic integration of this nutrient-dense oatmeal precursor with a completely additive-free fabrication strategy. To eliminate hazardous chemical cross-linkers, dehydrothermal (DHT) treatment was employed as a clean manufacturing route to stabilize the gelatin network. DHT processing involves thermal treatment under reduced pressure, which promotes dehydration and intermolecular condensation between the gelatin chains and the oxygen-rich surface groups of the CQDs without requiring external chemical reagents [17,18].

To the best of our knowledge, the systematic optimization of oat-derived CQDs incorporated into a DHT-treated gelatin hydrogel, paired with an all-encompassing evaluation of loading concentration, network architecture, degradation kinetics, and multi-technique mineral quantification, has not been previously reported.

The objective of this work was to develop and characterize a sustainable G/CQD hydrogel platform and isolate the exact CQD concentration providing the most favorable balance between structural stability, mechanical performance, swelling behavior, degradation resistance, and cytocompatibility. Crucially, the SBF experiments in this study were specifically designed to evaluate the physicochemical bioactivity and apatite-forming capability of the developed scaffolds at the material interface. They were not intended to establish osteogenic differentiation pathways or in vivo osteoconductivity, which require dedicated osteoblast cell lines and animal models in future investigations.

## 2. Results and Discussion

### 2.1. Structural and Morphological Characterizations of CQDs

**Figure 1:**
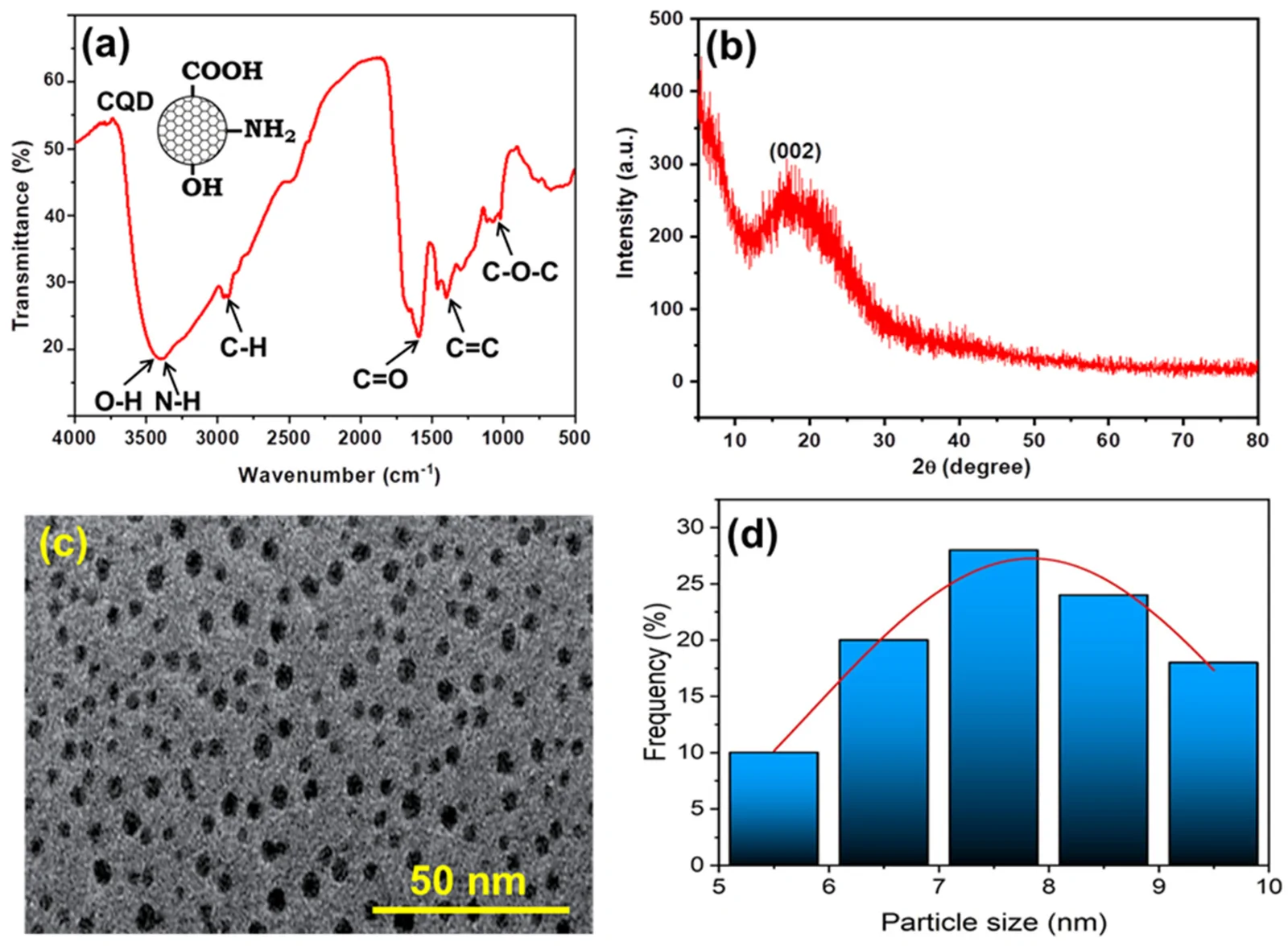
Structural and morphological characterization of oat-derived CQDs: (**a**) FTIR spectrum, (**b**) XRD pattern, (**c**) TEM micrograph, and (**d**) particle size distribution histogram of the synthesized CQDs.

The structural and morphological characteristics of the synthesized CQDs were investigated using FTIR, XRD, and TEM analyses, as presented in Figure 1. The FTIR spectrum (Figure 1a) exhibits a broad absorption band around 3200–3500 cm^−1^, which is attributed to O–H and N–H stretching vibrations, indicating the presence of hydroxyl and amine functional groups on the CQD surface [19]. The peaks observed near 1650 cm^−1^ and 1400 cm^−1^ correspond to C=O (amide or carboxyl) and C–N/C–O stretching vibrations, respectively [20]. Additionally, the inset image clearly displays the surface functional groups of CQDs. These functional groups confirm the successful surface functionalization of CQDs, which enhances their dispersibility and potential interaction with polymeric matrices.

The XRD pattern (Figure 1b) shows a broad diffraction peak centered around 2θ ≈ 20–25°, indexed to the (002) plane of graphitic carbon. The broad nature of this peak indicates the amorphous or turbostratic structure of CQDs, which is typical for carbon-based nanodots with low crystallinity [21].

The TEM image (Figure 1c) reveals that the CQDs are well-dispersed and quasi-spherical in shape with no significant aggregation [22]. The particles are uniformly distributed, indicating good stability of the synthesized nanodots. The particle size distribution (Figure 1d) shows that the CQDs have sizes predominantly in the range of 5–10 nm, with many particles centered around 7–8 nm. The relatively narrow and symmetric distribution, as confirmed by the Gaussian fitting, indicates uniform nucleation and controlled growth during the synthesis process [10,23].

### 2.2. Optical Properties and Colloidal Stability of CQDs

**Figure 2:**
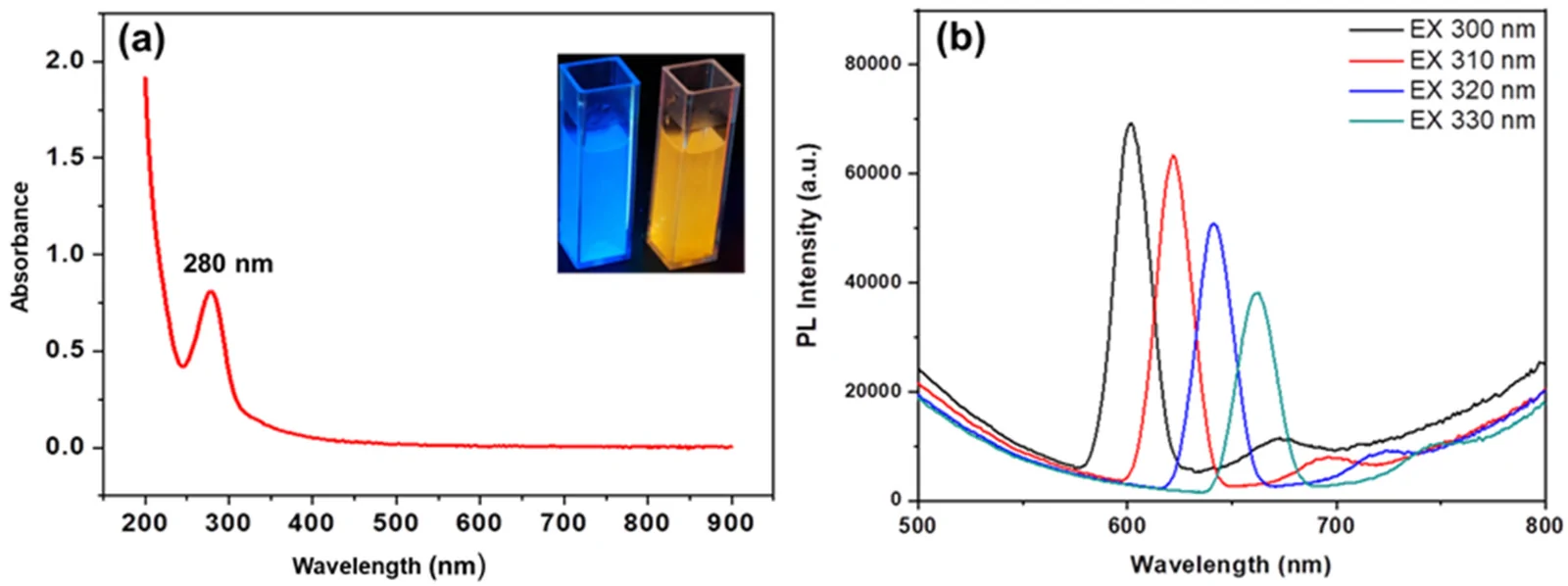
Optical characterization of oat-derived CQDs: (**a**) UV–Vis absorption spectrum and (**b**) photoluminescence (PL) emission spectra recorded at different excitation wavelengths; the inset shows the fluorescence emission of CQDs under UV irradiation.

The optical properties and electronic transitions of the synthesized CQDs were characterized using UV-Vis absorption and PL spectroscopy, as illustrated in Figure 2. The UV-Vis absorption spectrum in Figure 2a exhibits a well-defined primary absorption maximum at approximately 280 nm, which is characteristic of the π–π* electronic transitions originating from the sp^2^-hybridized carbon framework within the aromatic C=C clusters [19,24]. Additionally, a distinct absorption tail extending into the visible spectrum was observed, which is typically attributed to n–π* transitions associated with oxygen-containing surface functionalities such as carbonyl or carboxyl groups [25]. These spectrophotometric findings confirm the successful synthesis of carbonaceous nanostructures possessing diverse, surface-localized defect states.

The PL spectra presented in Figure 2b demonstrate a pronounced excitation-dependent emission profile, which serves as a hallmark of CQDs. As the excitation wavelength was incrementally tuned from 300 to 330 nm, the emission maxima underwent a systematic bathochromic shift, commonly referred to as a red shift, which was accompanied by a monotonic attenuation in PL intensity. This specific behavior suggests a high degree of structural and electronic heterogeneity within the sample, likely resulting from the distribution of varying sp2 domain sizes and the presence of diverse chemical environments that facilitate the stabilization of multiple emissive trap states [26].

The observation of peak quantum efficiency at the lower excitation wavelength of 300 nm indicates highly optimized radiative recombination pathways from the core states of the nanostructures. Conversely, the red-shift at longer wavelengths highlights the significant contribution of lower-energy surface states, a result that remains consistent with the oxygen-rich surface chemistry previously identified via FTIR analysis. Ultimately, the macroscopic fluorescence observed under UV irradiation (Figure 2a, inset) further corroborates the high optical activity and radiative efficiency of the synthesized CQDs, underscoring their potential for high-resolution sensing and advanced bioimaging applications [19,21,27].

Further insight into the colloidal stability of the synthesized CQDs was obtained from zeta potential measurements (Figure S1). The CQDs exhibited a highly negative zeta potential (−39.9 mV), indicating strong electrostatic repulsion between particles and, consequently, excellent colloidal stability in aqueous media [28]. This high stability can be attributed to the presence of abundant surface functional groups, such as hydroxyl and carboxyl moieties, as confirmed by FTIR analysis. In addition, oats were selected as a sustainable and nutrient-rich carbon precursor due to their high starch content and the presence of essential minerals (e.g., Fe and P), which facilitate efficient carbonization and surface passivation. The high negative zeta potential demonstrates that oat-derived CQDs possess superior stability compared to other biomass-derived CQDs reported from sources such as corn or lemon peel [14,29]. These characteristics make the prepared CQDs particularly suitable for stable dispersion and effective incorporation within hydrogel matrices.

### 2.3. Characterization of the G/CQD Nanocomposite Hydrogels

#### 2.3.1. Structural Analysis and Integration Mechanism

**Figure 3:**
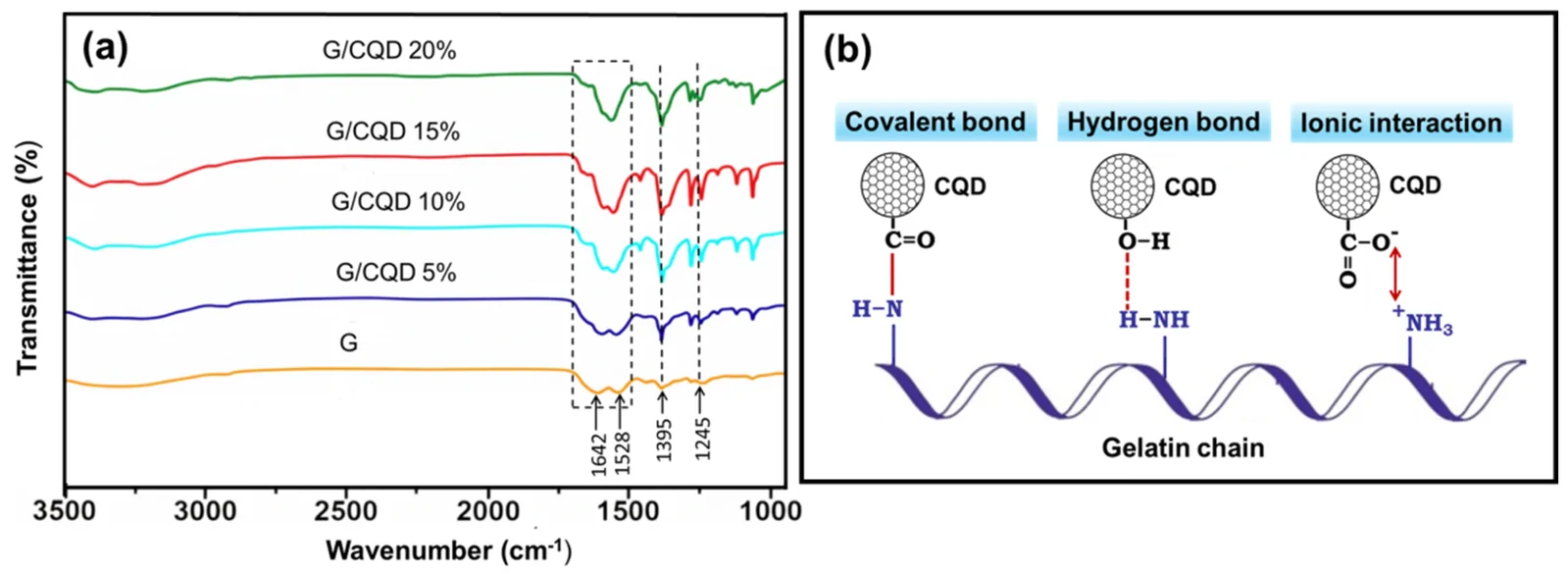
(**a**) FTIR spectra of pristine gelatin and G/CQD nanocomposite hydrogels with different CQD loadings; (**b**) schematic representation of the proposed integration mechanism, illustrating proposed covalent and non-covalent interactions between CQDs and the gelatin matrix.

Figure 3 presents the FTIR spectra of pristine gelatin and G/CQD hydrogel nanocomposites with varying CQD loadings (Figure 3a), together with a schematic illustration of the proposed integration mechanism (Figure 3b). The FTIR spectrum of gelatin exhibits a broad band at ~3469 cm^−1^, assigned to O–H stretching vibrations, along with characteristic peaks at ~2925 and 2850 cm^−1^ corresponding to C–H and CH_2_ stretching. The prominent band at ~1635 cm^−1^ is attributed to amide I (C=O stretching), while the bands at ~1453 and 1387 cm^−1^ correspond to amide II and III vibrations, respectively, reflecting the intrinsic triple-helix structure of gelatin [30,31].

Upon incorporation of CQDs, distinct spectral modifications were observed, particularly in the amide region. A shift in the amide I band from approximately 1635 to 1645 cm^−1^, accompanied by a change in band intensity, indicates modification of the chemical environment of the gelatin chains following CQD incorporation. These spectral changes are consistent with interactions between the functional groups present on the CQD surface and those of gelatin [32]. In particular, the oxygen-containing groups on the CQDs, including carboxyl and hydroxyl functionalities, may participate in hydrogen bonding and electrostatic interactions with the functional groups of gelatin [33]. The observed FTIR changes therefore support the successful integration of CQDs within the gelatin matrix and the formation of strong interfacial interactions. However, FTIR spectroscopy alone cannot definitively prove the formation of new covalent amide bonds; such confirmation would require complementary techniques like XPS, Raman spectroscopy, solid-state NMR, or chemical crosslinking analysis [31].

In addition to FTIR analysis, the structural organization of the hydrogels was further investigated by XRD (Figure S2). Pristine gelatin exhibited a broad diffraction halo centered at approximately 2θ = 20°, characteristic of its predominantly amorphous structure. Following the incorporation of CQDs, all G/CQD nanocomposite hydrogels retained the broad amorphous diffraction profile without the appearance of distinct crystalline peaks, indicating that the introduction of CQDs did not induce phase separation or crystallization within the gelatin matrix [34,35]. However, a slight broadening and reduction in peak intensity were observed with increasing CQD loading, suggesting enhanced intermolecular interactions and modification of the molecular packing of gelatin chains [36].

#### 2.3.2. Morphology and Pore Architecture

**Figure 4:**
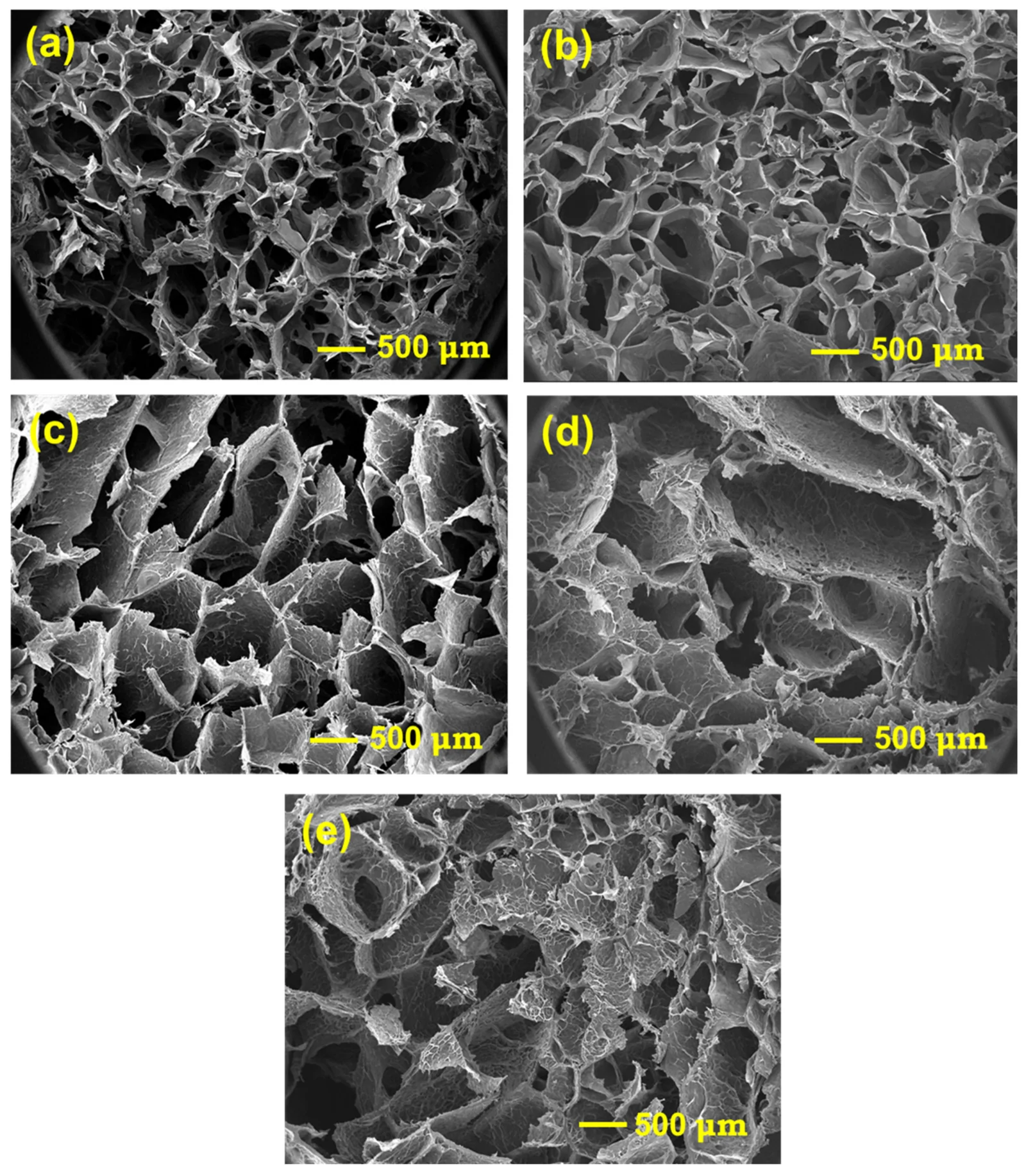
SEM micrographs illustrating the morphological evolution of pristine gelatin and G/CQD nanocomposite hydrogels with increasing CQD loading: (**a**) pristine gelatin hydrogel (G, 0% CQDs), (**b**) G/CQD 5%, (**c**) G/CQD 10%, (**d**) G/CQD 15%, and (**e**) G/CQD 20%. All samples exhibit interconnected porous architecture with progressive structural densification upon CQD incorporation.

The microstructure of the prepared hydrogels was examined by SEM after freeze-drying to evaluate the internal pore architecture of the networks. All samples exhibited highly interconnected porous structures with pore sizes in the micrometer range, which is characteristic of freeze-dried hydrogel systems. The pore size distribution was quantified using ImageJ software, and representative micrographs are presented in Figure 4. As shown in Figure 4a, the pristine gelatin hydrogel displays a well-defined, highly porous, honeycomb-like morphology with large and uniformly distributed pores [37,38]. The average pore size was determined to be approximately 330 ± 46 μm, reflecting a loosely crosslinked polymer network with substantial free volume.

Upon incorporation of CQDs, a pronounced alteration in internal morphology is observed. At 5% CQD loading (Figure 4b), the hydrogel structure becomes noticeably more compact, accompanied by a sharp reduction in pore size to 131 ± 52 μm. This significant decrease suggests that even at low concentrations, CQDs act as effective nano-crosslinking agents, reinforcing the gelatin network [32,35].

With further increasing CQD content (10–20%, Figure 4c–e), a progressive densification of the network is evident. The pore structure becomes increasingly constricted and less regular, while the pore walls appear thicker and more rigid. This evolution reflects an increase in crosslinking density, leading to a tighter and more compact three-dimensional structure. At higher CQD loading, minor irregularities in pore morphology may also arise, likely due to partial nanoparticle aggregation or saturation of available interaction sites [39].

The reduction in pore size and overall porosity can be attributed to strong intermolecular interactions between CQDs and gelatin chains. The presence of abundant oxygen-containing functional groups (–OH and –COOH) on the CQD surface promotes hydrogen bonding and electrostatic interactions with the amino groups of gelatin. These interactions restrict polymer chain mobility and enhance network packing, resulting in a more compact internal structure [31,40].

From a biomaterial perspective, pore architecture represents a crucial balance between structural integrity and biological accessibility. An interconnected porous structure is essential for facilitating fluid transport, nutrient diffusion, cell attachment, and subsequent cellular infiltration, whereas excessive pore compaction could restrict these processes. Therefore, the reduction in pore size observed in the present study should not be interpreted as universally beneficial for bone regeneration; rather, it reflects enhanced network compactness and mechanical stability resulting from CQD incorporation [41]. Determining whether the resulting pore architecture is fully optimized for osteogenic cell infiltration and tissue ingrowth would require dedicated osteoblast differentiation and in vivo investigations, which are beyond the scope of the present study.

#### 2.3.3. Mechanical Properties

**Figure 5:**
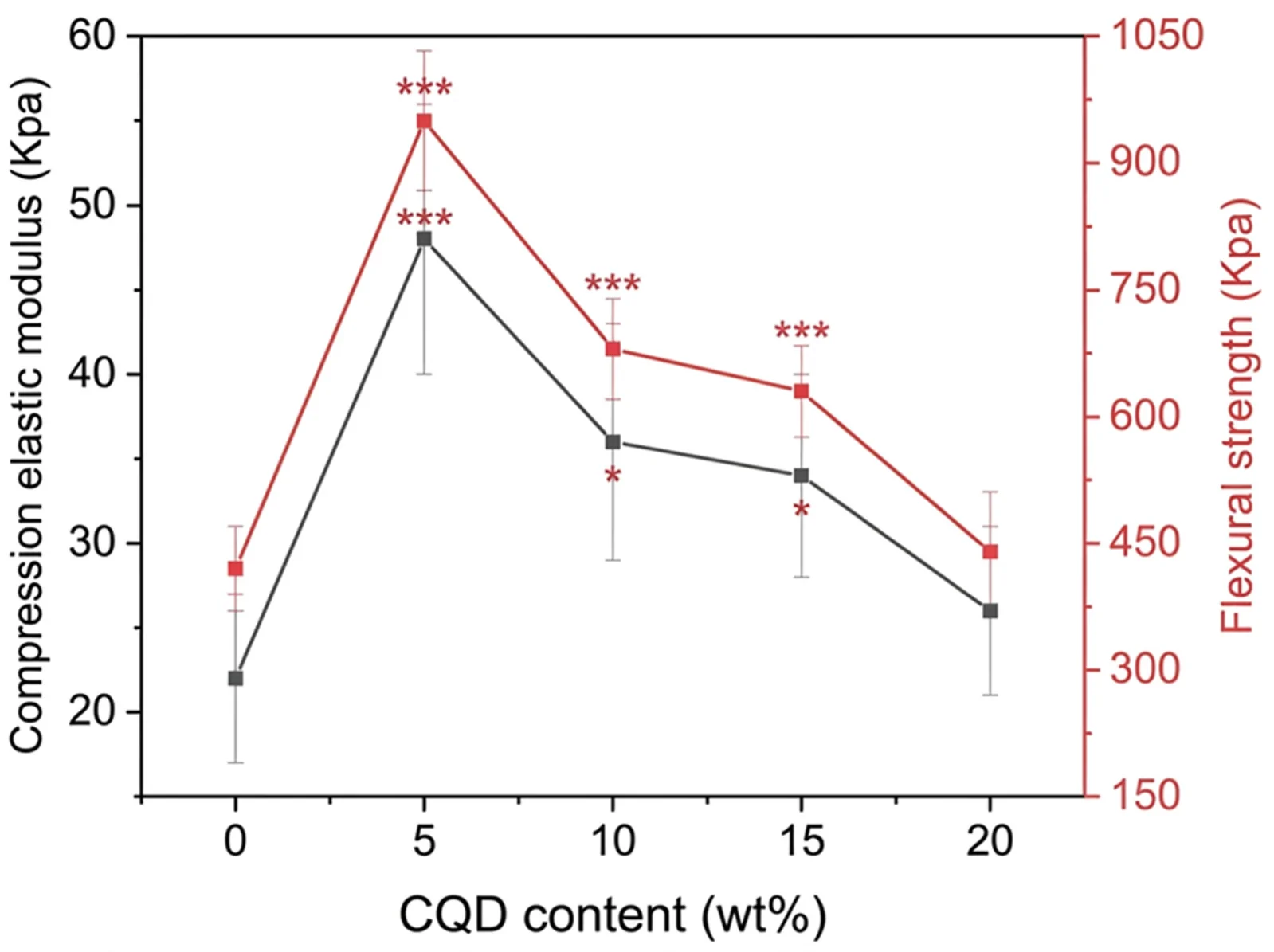
Mechanical performance of G/CQD nanocomposite hydrogels as a function of CQD loading, illustrating the variation in compression elastic modulus (left axis) and flexural strength (right axis) with increasing CQD content. Data are presented as mean ± SD (*n* = 5). Statistical significance relative to the pristine gelatin control group was assessed using one-way ANOVA: * *p* < 0.05 and *** *p* < 0.001.

The mechanical performance of the prepared hydrogels was evaluated through compression elastic modulus and flexural strength measurements, and the results are summarized in Figure 5. As shown in Figure 5, the incorporation of CQDs significantly influenced the mechanical properties of the gelatin hydrogel. The pristine gelatin hydrogel exhibited a relatively low compression elastic modulus of 22 kPa and a flexural strength of 420 kPa, reflecting the soft and loosely crosslinked structure of the gelatin network [17,42].

Upon the addition of CQDs, a notable improvement in mechanical properties was observed. The hydrogel containing 5% CQDs showed the highest mechanical performance, with the compression elastic modulus increasing to 48 kPa and the flexural strength reaching 950 kPa. This enhancement can be attributed to the effective interaction between CQDs and gelatin chains, where the oxygen-containing functional groups on the CQD surface form hydrogen bonding and electrostatic interactions with gelatin [17,43]. These interactions act as additional physical crosslinking points, reinforcing the polymer network and improving its mechanical stability.

However, further increasing the CQD content beyond 5% resulted in a gradual decrease in both compression modulus and flexural strength. This behavior may be associated with partial aggregation of CQDs at higher concentrations, which can reduce the efficiency of stress transfer within the hydrogel matrix and slightly disrupt the uniformity of the network structure [25,32].

### 2.4. Swelling Behavior

**Figure 6:**
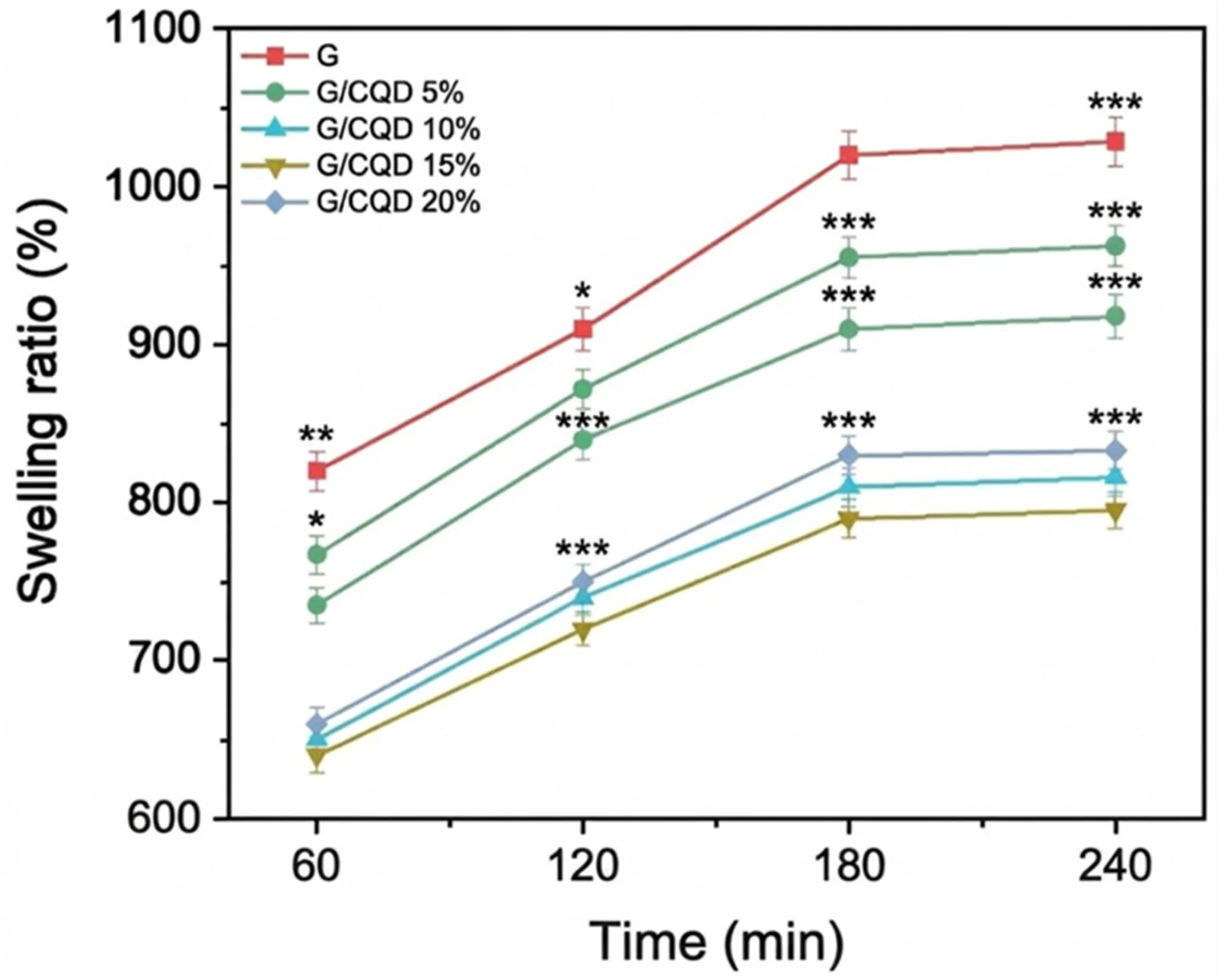
Swelling behavior of pristine gelatin and G/CQD nanocomposite hydrogels in distilled water, showing the variation in swelling ratio as a function of immersion time. Data are presented as mean ± SD (*n* = 5). Statistical significance relative to the pristine gelatin control group was assessed using one-way ANOVA: * *p* < 0.05, ** *p* < 0.01, and *** *p* < 0.001.

As shown in Figure 6, the swelling behavior of pristine gelatin hydrogel and G/CQD nanocomposite hydrogels (5–20%) was investigated in aqueous medium for up to 240 min. All samples exhibited a gradual increase in swelling ratio with immersion time until approaching equilibrium after approximately 180–240 min. This behavior is typical of diffusion-controlled water uptake in hydrogel networks, where water molecules progressively penetrate the porous polymer structure until osmotic and elastic forces reach equilibrium.

The pristine gelatin hydrogel displayed the highest swelling ratio throughout the experiment, reaching approximately 1029% after 240 min. This high water absorption can be attributed to the presence of abundant hydrophilic functional groups in gelatin, such as amide, carboxyl, and hydroxyl groups, which readily interact with water molecules through hydrogen bonding [31,44].

Incorporation of CQDs into the gelatin matrix resulted in an overall decrease in swelling capacity compared with pristine gelatin. This behavior can be attributed to the additional intermolecular interactions established between the oxygen-containing functional groups on the CQD surfaces and the functional groups of gelatin. These interactions can act as secondary physical crosslinking points within the polymer network, restricting gelatin-chain mobility and reducing the free volume available for water accommodation.

In addition, the resulting increase in network compactness can reduce the size and accessibility of water-diffusion pathways. Consequently, water molecules experience greater resistance to penetration and redistribution within the polymer network, leading to the observed reduction in swelling capacity. Thus, the lower swelling of the CQD-containing hydrogels is consistent with the formation of a more compact and structurally constrained gelatin network [35,40,45].

### 2.5. In Vitro Degradation Behavior of G/CQD Nanocomposite Hydrogels

**Figure 7:**
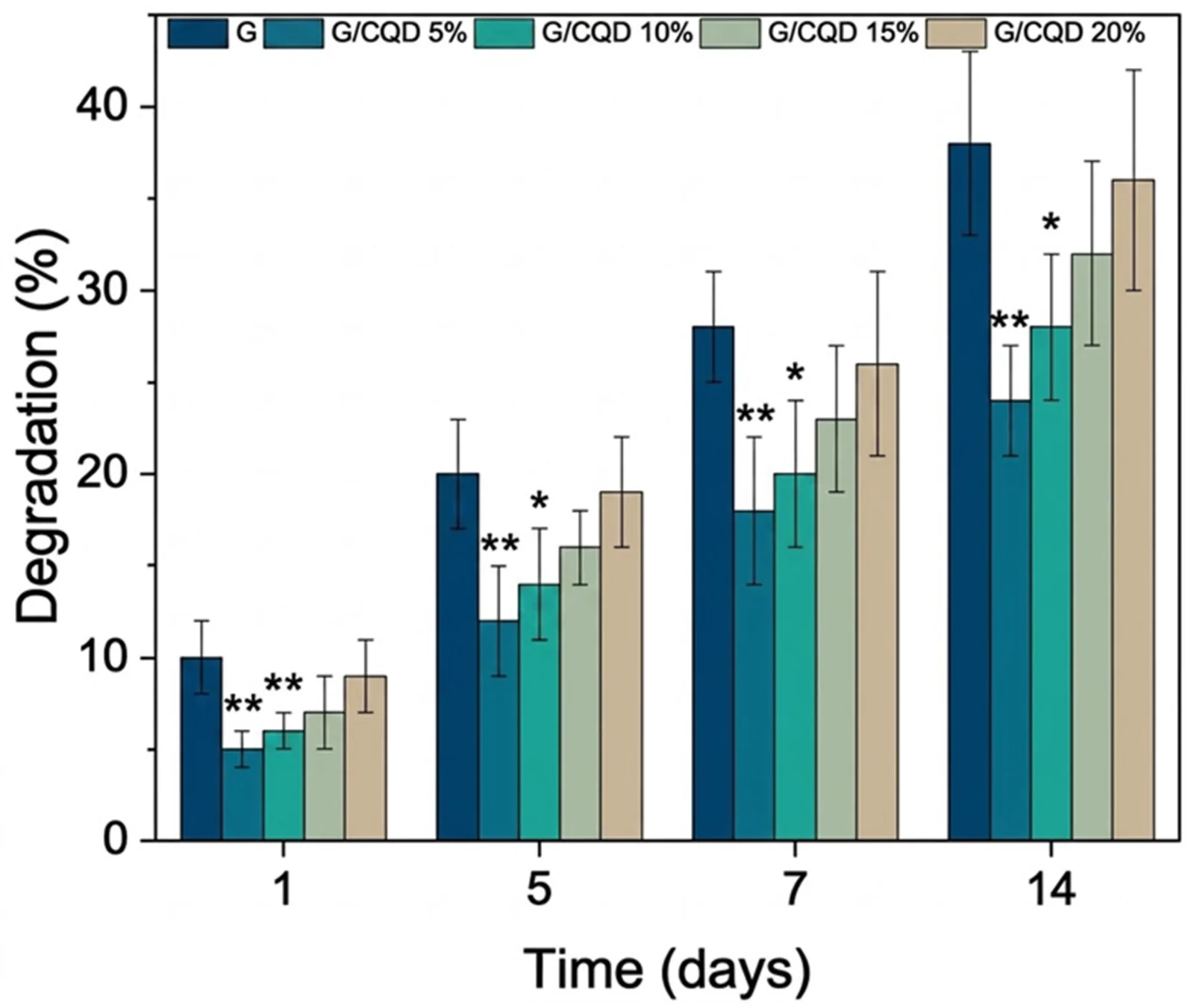
In vitro degradation profiles of pristine gelatin (G) and G/CQD nanocomposite hydrogels over a 14-day incubation period. Data are presented as mean ± SD (*n* = 5). Statistical significance relative to the pristine gelatin control group was assessed using one-way ANOVA: * *p* < 0.05 and ** *p* < 0.01.

The in vitro degradation behavior of the pristine gelatin hydrogel and the G/CQD nanocomposite hydrogels was investigated over a period of 14 days, and the results are presented in Figure 7. As observed, all hydrogel samples exhibited a gradual increase in mass loss with increasing incubation time, indicating progressive degradation of the polymer network in the aqueous medium.

The pristine gelatin hydrogel exhibited approximately 40% mass loss after 14 days of incubation, demonstrating the highest degradation rate throughout the experiment. This behavior can be attributed to the relatively loose network structure of unreinforced gelatin and the presence of abundant hydrophilic functional groups that facilitate rapid water penetration and subsequent hydrolytic cleavage of the polymer chains [31,46]. Consequently, the pristine gelatin matrix gradually lost its structural integrity during the incubation period.

In contrast, the incorporation of CQDs into the gelatin matrix substantially reduced the mass loss of the hydrogels, with the G/CQD 5 wt% formulation restricting mass loss to approximately 24% after 14 days. Thus, the integration of 5 wt% CQDs resulted in an approximate reduction of 16% in mass loss relative to the pristine gelatin control. This enhanced resistance to degradation is driven by strong interfacial interactions between the CQD surfaces and the gelatin chains, where oxygen-containing functional groups (such as carboxyl and hydroxyl moieties) form dense hydrogen bonding networks and electrostatic complexes. These non-covalent interactions restrict biopolymer chain mobility and increase the compactness of the hydrogel framework, thereby reducing water accessibility to the hydrophilic backbones and slowing down network mass loss [47,48].

At higher CQD loadings (10–20%), the degradation rate slightly increased compared to the 5 wt% formulation. This behavior may be associated with the partial aggregation of CQDs at higher concentrations, which introduces structural heterogeneity and defects within the hydrogel network [49]. Such controlled and tunable degradation kinetics are highly desirable for biomaterial applications, particularly in tissue engineering, where scaffolds are expected to maintain an effective structural template during the critical early stages of tissue regeneration while gradually degrading as neo-tissue integration proceeds [50,51].

Although the internal network density was not quantitatively determined in the present study, the combined structural, swelling, mechanical, and degradation datasets provide cohesive evidence for a clear qualitative relationship between matrix compactness and overall structural stability. The integration of biomass-derived CQDs systematically produced a more compact pore architecture, controlled swelling kinetics, enhanced mechanical performance, and restricted mass loss. Taken together, these macroscopic observations firmly validate that CQDs serve as effective interfacial stabilizing agents that restrict polymer chain mobility and reinforce the underlying biopolymer framework [32].

### 2.6. Biological Safety and Cytocompatibility of G/CQD Scaffolds

**Figure 8:**
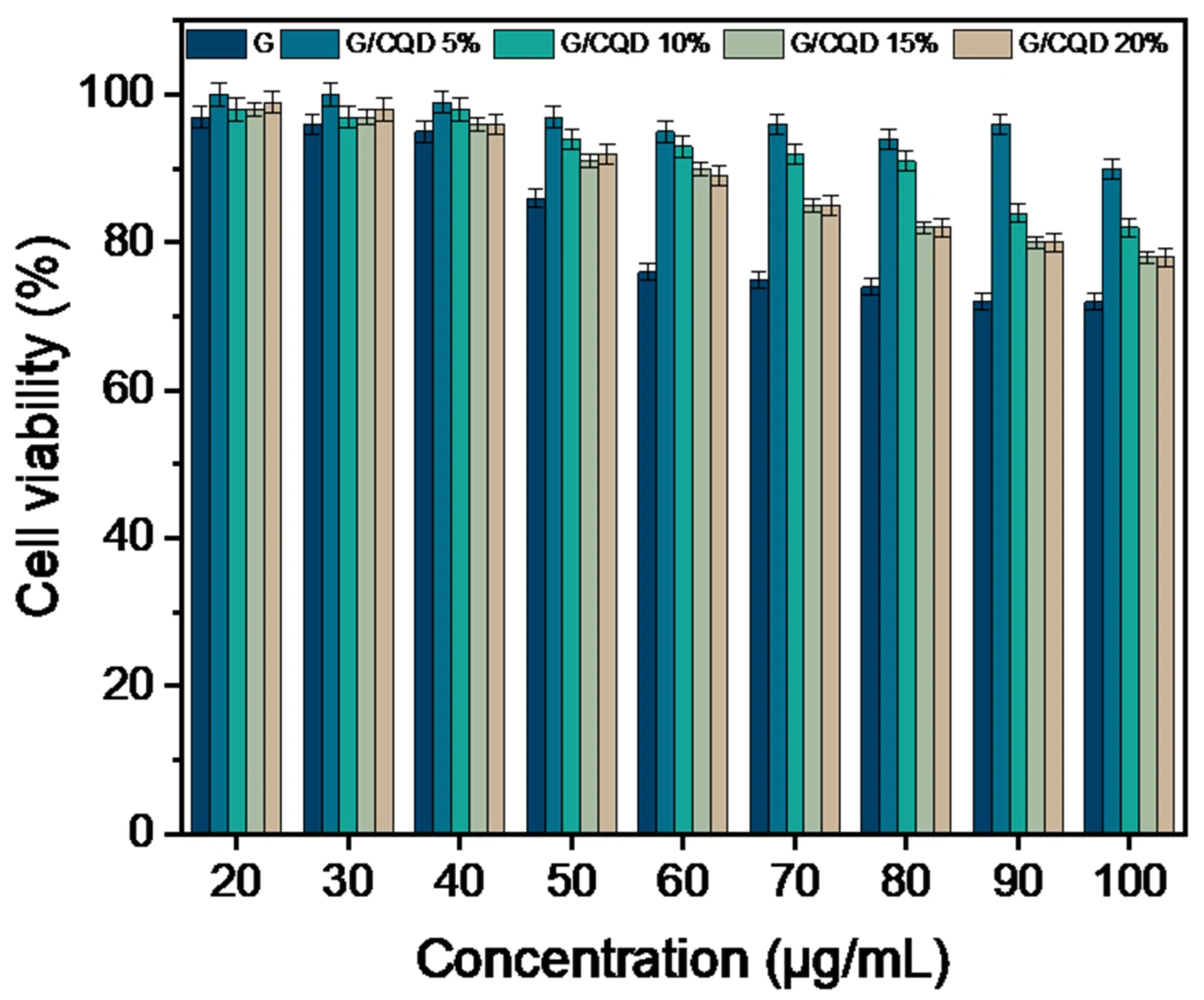
Comparative cell viability (%) of Human Skin Fibroblasts (HSFs) following 24 h exposure to pristine gelatin (G) and G/CQD nanocomposite hydrogel scaffold extracts across varying sample concentrations (20–100 µg/mL). Data are presented as mean ± SD (*n* = 5). Statistical significance relative to the pristine gelatin control group was assessed at each concentration using one-way ANOVA, with *p* < 0.001 considered highly statistically significant.

The cytocompatibility of the prepared G/CQD hydrogels was evaluated by measuring cell viability at different CQD concentrations, as presented in Figure 8. Overall, the investigated samples exhibited high cell viability, indicating favorable preliminary cytocompatibility under the experimental conditions employed.

The pristine gelatin hydrogel showed excellent biocompatibility, with cell viability exceeding 90%, which is consistent with the well-known biocompatible nature of gelatin. Upon incorporation of CQDs, a slight variation in cell viability was observed depending on the CQD content. At the optimal 5% CQD concentration, the hydrogels maintained high viability levels comparable to the pristine gelatin, suggesting that the incorporation of CQDs does not adversely affect cellular response [35].

At higher CQD loadings (10–20%), a marginal decrease in cell viability may be observed; however, the values remain well within the acceptable ISO standard threshold for biocompatible materials (≥70%), indicating that the nanocomposites retain their cytocompatibility. This slight reduction can be attributed to increased nanoparticle content, which may influence cell–material interactions or induce mild cellular stress at elevated concentrations [52,53].

The overall favorable cytocompatibility can be attributed to the inherent biocompatibility of gelatin combined with the stable surface chemistry of CQDs, which are rich in oxygen-containing functional groups. Additionally, the homogeneous dispersion of CQDs within the hydrogel matrix likely minimizes localized nanoparticle aggregation, thereby reducing potential cytotoxic effects [49].

Based on the combined physicochemical and biological results, the incorporation of CQDs at 5% provides the most balanced performance among the formulations investigated. At this concentration, the hydrogel exhibits a favorable combination of structural integrity, controlled pore architecture, and high cytocompatibility, without the potential drawbacks associated with higher CQD loadings such as excessive network densification or a slight reduction in cell viability.

It should be noted that HSF cytocompatibility provides an initial assessment of the biological safety of the hydrogel but does not establish osteogenic activity or osteoconductivity. The present biological evaluation was therefore considered a preliminary cytocompatibility assessment, while the bone-related functionality of the materials was investigated strictly through their SBF-induced mineralization behavior. Dedicated osteogenic cell studies using appropriate preosteoblast or osteoblast-like models remain an essential requirement for future investigations to evaluate cell differentiation and targeted bone regenerative activity.

Accordingly, the G/CQD 5% nanocomposite hydrogel was selected as the optimal composition for further evaluation of bioactivity in SBF. This selection is based on its ability to maintain a well-integrated network structure while preserving desirable biological performance, making it a highly suitable template for subsequent in vitro biomineralization studies.

### 2.7. In Vitro Bioactivity and Apatite Formation

**Figure 9:**
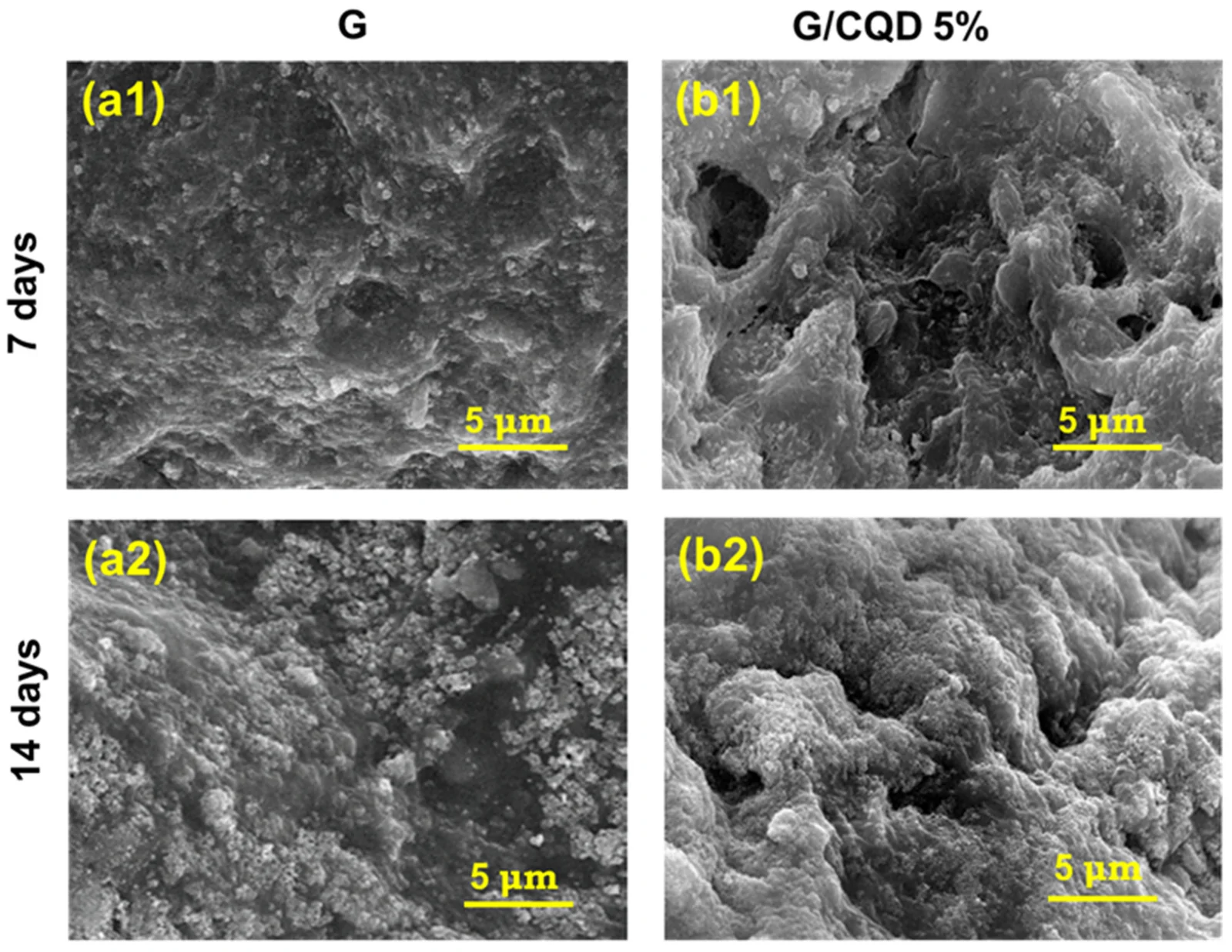
SEM micrographs of pristine gelatin (G) and G/CQD 5% nanocomposite hydrogels after immersion in SBF for 7 and 14 days. (**a1**,**a2**) Pristine gelatin showing limited mineral deposition; (**b1**,**b2**) G/CQD 5% showing enhanced surface mineral deposition and formation of an apatite-like layer following SBF immersion.

The in vitro bioactivity of the prepared hydrogels was evaluated by monitoring apatite formation after immersion in SBF for 7 and 14 days, as presented in Figure 9. SEM micrographs reveal clear differences in surface morphology between pristine gelatin and the G/CQD 5% nanocomposite, highlighting the critical role of CQDs in enhancing biomineralization.

For pristine gelatin (Figure 9(a1,a2)), only limited mineral deposition is observed after both immersion periods. The surface remains relatively smooth with sparse and discontinuous particulate formation, indicating a low nucleation capability. This behavior is typical of pure gelatin matrices, which lack sufficient active sites to effectively induce apatite growth despite their hydrophilic nature [54].

In contrast, the G/CQD 5% nanocomposite (Figure 9(b1,b2)) exhibits significantly enhanced bioactivity. After 7 days, the surface becomes rougher with the appearance of numerous nucleation clusters, indicating the initiation of apatite formation. After 14 days, a dense and continuous layer of cauliflower-like apatite structures fully covers the hydrogel surface. This morphology is characteristic of bone-like HA, suggesting an inherent biomimetic mineralization capacity for the nanocomposite and indicating its potential as a scaffold for bone tissue engineering applications.

This enhanced apatite formation can be attributed to the presence of CQDs, which introduce abundant oxygen-containing functional groups (–COOH and –OH) on the hydrogel surface. These groups act as effective nucleation sites by attracting Ca^2+^ ions from the SBF solution, followed by interaction with phosphate ions, ultimately leading to apatite crystallization. In addition, the negatively charged surface of CQDs further facilitates electrostatic attraction of calcium ions, accelerating the nucleation process [55,56].

Furthermore, the homogeneous dispersion of CQDs within the gelatin matrix ensures uniform distribution of these active sites, resulting in a more consistent and denser mineral layer compared to pristine gelatin. The synergistic effect of surface chemistry and improved microstructure, therefore, significantly enhances the bioactivity of the hydrogel [57].

Overall, these results confirm that the incorporation of CQDs not only modifies the physicochemical properties of gelatin hydrogels but also plays a key role in promoting rapid and dense apatite formation, making the G/CQD 5% nanocomposite a promising candidate for bone tissue engineering applications. The structural and elemental evolution of the G/CQD 5% hydrogel following 14 days of immersion in SBF suggests a promising capacity for biomineralization, indicating the material’s potential bioactivity.

**Figure 10:**
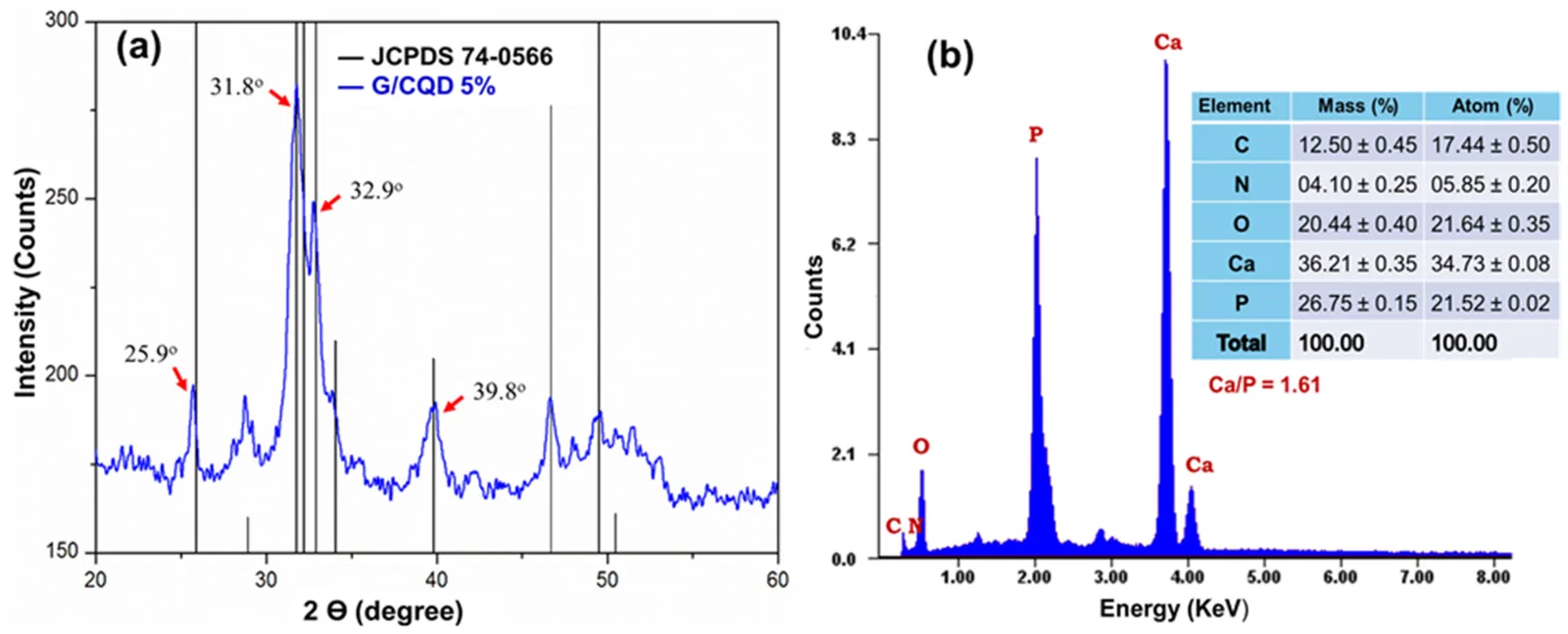
(**a**) XRD pattern of the mineralized G/CQD 5% hydrogel after 14 days of immersion in SBF, showing diffraction features characteristic of HA; (**b**) EDX spectrum of the mineralized surface showing the presence of Ca and P. The calculated Ca/P molar ratio was approximately 1.61.

As shown in the XRD pattern of the mineralized surface after 14 days of immersion in SBF (Figure 10a), distinct diffraction peaks are observed that correspond well to the characteristic hexagonal phase of HA, in agreement with JCPDS card no. 74-0566. The appearance of characteristic reflections at 2θ values of 25.9°, 31.8°, 32.9°, and 39.8° confirms that the mineral layer is not merely an amorphous precipitate but a well-developed crystalline phase [58,59]. The notable intensity of the (211) and (300) peaks suggests a high degree of crystallinity, while the slight broadening of the peaks indicates the formation of nanocrystalline HA, which is structurally analogous to the inorganic component of natural bone tissue [60].

The chemical integrity of this mineral layer is further substantiated by the EDX spectrum and its corresponding quantitative analysis (Figure 10b). The dominance of calcium (Ca) and phosphorus (P) peaks, alongside oxygen (O), confirms the deposition of a calcium-phosphate-based mineral. The measured Ca/P molar ratio of 1.61 is slightly lower than the stoichiometric Ca/P ratio of 1.67 characteristic of natural HA. This difference suggests the formation of a mildly calcium-deficient apatite phase. Calcium-deficient apatites generally exhibit greater solubility and a higher tendency toward dissolution and reprecipitation compared with highly stoichiometric HA. Such behavior may be advantageous in bone-related biomaterials because a more dynamically resorbable mineral phase can participate in mineral exchange and remodeling processes [61,62]. Nevertheless, the present SBF experiment demonstrates only the formation of an apatite-like mineral layer under acellular conditions and should not be interpreted as direct evidence of enhanced bone regeneration.

Beyond the mineral components, the EDX analysis successfully detected carbon (C) and nitrogen (N) signals, which originate from the gelatin polypeptide chains and the embedded CQDs. The simultaneous detection of the organic matrix and the thick mineral crust proves that the HA layer is intimately integrated with the hydrogel network rather than being a loose surface deposit. This interfacial bonding is a direct result of the oxygen-containing functional groups on the CQD surfaces, which serve as high-affinity nucleation sites. These sites effectively lower the interfacial energy required for mineral precipitation, trapping Ca^2+^ ions from the SBF and subsequently anchoring the PO43- groups to initiate crystal growth [63].

The combined identification of crystalline phases via XRD and stoichiometric verification via EDX indicates that the incorporation of 5% CQDs enhances the biomineralization potential of the gelatin matrix. The formation of an apatite-like layer with a Ca/P ratio approaching that of natural bone within 14 days suggests that these nanocomposites may serve as effective templates for mineral deposition. This surface-mineralized architecture offers a biomimetic interface that could support further biological evaluation. While these results show that the CQDs facilitate the nucleation process, additional studies using osteogenic cell lines and bone-specific markers are required to fully assess the scaffold’s osteoconductive properties and its suitability for bone regeneration applications.

## 3. Conclusions

In this study, oat-derived carbon quantum dots were successfully synthesized through a green hydrothermal process and incorporated into DHT-treated gelatin hydrogels to develop sustainable nanocomposite scaffolds. The incorporation of CQDs significantly enhanced the structural, morphological, mechanical, swelling, and degradation properties of the biopolymer matrix. Among the investigated formulations, G/CQD 5% exhibited the most favorable overall balance of structural integrity, mechanical performance, and controlled swelling kinetics. Notably, this optimized formulation reduced the network mass loss after 14 days of incubation to approximately 24%, compared with 40% for the unreinforced pristine gelatin control. Furthermore, immersion in simulated body fluid resulted in rapid and pronounced mineral deposition across the G/CQD 5% interface. Advanced structural, morphological, and elemental characterization using SEM, XRD, and EDX confirmed the formation of an apatite-like crystalline mineral layer with a biomimetic Ca/P molar ratio of approximately 1.61 after 14 days. These findings demonstrate that the composite can support in vitro apatite nucleation and surface mineral deposition under acellular conditions. The CQD-containing hydrogels also exhibited favorable preliminary cytocompatibility with human skin fibroblasts, with cell viability remaining above 90% under the experimental conditions. Overall, incorporating biomass-derived CQDs appears to be an effective strategy for tailoring the physicochemical stability and interfacial mineralization behavior of gelatin hydrogels. The combination of renewable-precursor-based CQD synthesis and additive-free DHT processing provides a clean, toxic-reagent-free manufacturing pathway for developing functional gelatin-based biomaterials. However, the present study focused primarily on material design, physicochemical characterization, baseline cytocompatibility, and acellular in vitro mineralization in SBF. The current findings do not establish osteogenic differentiation, osteoconductivity, or in vivo bone regeneration. Future investigations should therefore evaluate the developed scaffolds using dedicated osteogenic cell models, including preosteoblast or osteoblast-like cell lines, together with specific osteogenic differentiation markers and long-term proliferation assessments. Subsequent in vivo studies will also be necessary to comprehensively evaluate bone architecture formation, vascularization, host immune responses, tissue integration, and the coordinated processes of scaffold degradation and remodeling. Together, these investigations will provide the evidence needed to determine the translational potential of the developed G/CQD scaffolds for bone-repair applications.

## 4. Materials and Methods

### 4.1. Materials

Oats were obtained from a local commercial source and used as the carbon precursor for CQD synthesis. Type B gelatin from bovine skin (catalog number: G9382, ∼225 g Bloom; protein content >70%, and molecular weight: 50 kDa), hydrochloric acid (HCl), sodium hydroxide (NaOH), tris(hydroxymethyl) aminomethane (NH_2_C(HOCH_2_)_3_), dipotassium hydrogen phosphate trihydrate (K_2_HPO_4_·3H_2_O), potassium chloride (KCl), calcium chloride (CaCl_2_), disodium hydrogen phosphate (Na_2_HPO_4_), sodium chloride (NaCl), and sodium sulfate (Na_2_SO_4_) were purchased from Sigma Aldrich (St. Louis, MO, USA). Magnesium chloride hexahydrate (MgCl_2_·6H_2_O) and sodium hydrogen carbonate (NaHCO_3_) were obtained from El Nasr Pharmaceutical Chemicals Company (Kalyobea, Egypt). Potassium dihydrogen phosphate (KH_2_PO_4_) was purchased from Loba Chemie Pvt. Ltd. (Mumbai, India). Dimethyl sulfoxide (DMSO) was obtained from Loba Chemie Pvt. Ltd. (Mumbai, India). Dulbecco’s Modified Eagle Medium (DMEM) and fetal bovine serum (FBS) were purchased from Lonza (Basel, Switzerland). 3-(4,5-dimethylthiazol-2-yl)-2,5-diphenyltetrazolium bromide (MTT) was obtained from Sigma Aldrich (St. Louis, MO, USA). Phosphate-buffered saline (PBS) and simulated body fluid (SBF) were prepared according to standard protocols using analytical grade reagents. Deionized water was used for the preparation of all aqueous solutions. All chemicals were used as received without further purification.

### 4.2. Synthesis of CQDs

CQDs were synthesized using the green hydrothermal method [64]. Oat powder was first washed thoroughly with deionized water and dried at 80 °C for 30 min. The dried material was ground to obtain a fine powder. For CQD synthesis, 2 wt% oat powder was dispersed in deionized water under magnetic stirring for 30 min to obtain a homogeneous suspension. The suspension was transferred into a Teflon-lined stainless steel autoclave and heated at 180 °C for 8 h. After natural cooling to room temperature, the resulting brown solution was centrifuged at 14,000 rpm for 30 min to remove larger carbonized residues. The supernatant was filtered through a 0.22 μm membrane filter to eliminate remaining particulates. The purified solution was lyophilized to obtain solid CQDs. The production yield was calculated based on the mass of dried CQDs per unit volume of precursor solution and was determined to be 3.3 mg mL^−1^.

### 4.3. Preparation of Gelatin and G/CQD Nanocomposite Hydrogels

**Figure 11:**
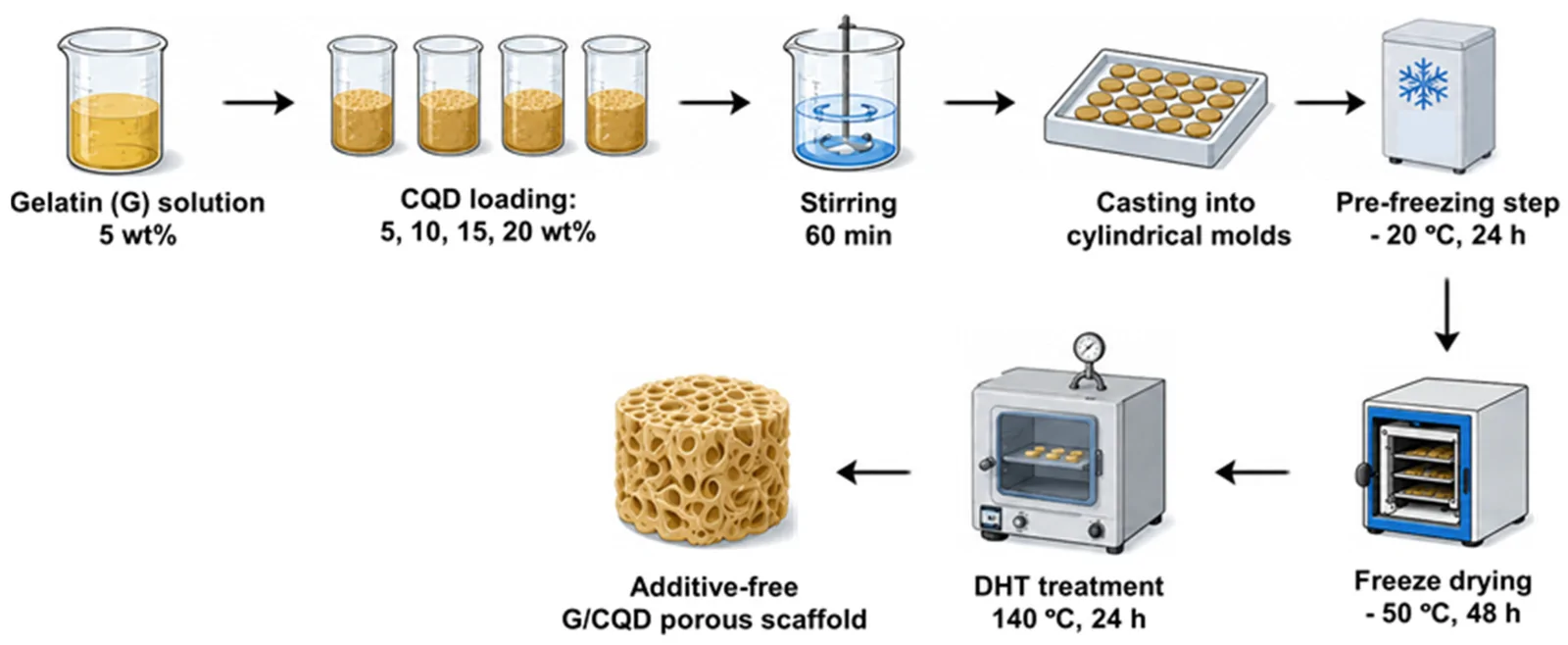
Schematic illustration of the preparation of G/CQD nanocomposite hydrogels using a combination of freeze-drying and DHT treatment.

Gelatin hydrogels and G/CQD nanocomposite hydrogels were prepared via physical crosslinking, using a combination of freeze-drying and DHT treatment, as illustrated in Figure 11. Gelatin (1 g) was dissolved in 20 mL of deionized water (5 wt%) under constant magnetic stirring at 35 °C until a clear, homogeneous solution was obtained. Predetermined volumes of the oat-derived CQD dispersion were added to the gelatin solution to achieve nanoparticle loadings of 5, 10, 15, and 20 wt% relative to the dry weight of gelatin. The resulting mixtures were stirred for 60 min to ensure uniform distribution of the CQDs within the polymer matrix. The solutions were then cast into cylindrical molds and pre-frozen at −20 °C for 24 h, followed by primary lyophilization in a freeze-dryer at −50 °C for 48 h to produce highly porous, non-crosslinked foam structures. To induce physical crosslinking without the use of chemical reagents, the freeze-dried scaffolds were subjected to DHT treatment. The scaffolds were placed in a vacuum oven at 140 °C under a vacuum pressure of <100 mTorr for 24 h. This process facilitates a condensation reaction between the carboxyl (–COOH) groups of acidic amino acid residues and the amino (–NH_2_) groups of basic amino acid residues in the gelatin chains, resulting in the formation of zero-length intermolecular amide bonds. After the DHT treatment, the scaffolds were cooled to room temperature in a desiccator and stored under dry conditions until further characterization.

### 4.4. Characterization of CQDs and G/CQD Nanocomposite Hydrogels

#### 4.4.1. Fourier Transform Infrared (FTIR) Spectroscopy

FTIR spectra for the prepared samples were recorded using the Perkin-Elmer FTIR spectrometer Spectrum Two (Perkin-Elmer, Waltham, MA, USA). The spectra were recorded throughout the range from 500 cm^−1^ to 4000 cm^−1^ at a resolution of 4 cm^−1^ with 32 scans per sample for spectroscopic identification of the functional groups present in the CQDs particles and G/CQD nanocomposite hydrogels. The samples were prepared using the KBr disc technique, in which the powder samples (5 mg) were compressed with KBr (200 mg) under hydraulic pressure to form a disk with a diameter of 13 mm.

#### 4.4.2. X-Ray Diffraction (XRD)

Structural analysis of the CQDs particles and G/CQD nanocomposite hydrogels was accomplished via XRD measurements using a Diffractometer (XRD-7000, Shimadzu, Kyoto, Japan). The X-ray beam was Cu-Kα radiation (λ = 0.15418 nm), operated at 40 kV and 38 mA. The XRD pattern was recorded in the 2θ range from 0° to 80° with a scanning rate that was 5° per min.

#### 4.4.3. Energy Dispersive X-Ray (EDX) Spectroscopy

The elemental composition and mineral distribution of the G/CQD nanocomposite hydrogels were analyzed using EDX spectroscopy. To ensure high accuracy for the determination of the Ca/P molar ratio, the system was calibrated using a standard hydroxyapatite reference material prior to analysis. The samples were analyzed in an uncoated state at an accelerating voltage of 15 kV with a high vacuum to minimize background interference. Data acquisition was performed for 60 s per site to ensure sufficient signal-to-noise ratios for calcium and phosphorus peaks. Quantitative elemental weight percentages were calculated using the ZAF correction method (Atomic number, Absorption, and Fluorescence) to account for matrix effects and sample density.

#### 4.4.4. Transmission Electron Microscopy (TEM)

The morphology and grain size of CQD particles were analyzed using TEM (model JEOL-JEM-100CX, Tokyo, Japan) operating at an accelerating voltage of 80 kV. A few drops of CQDs powder (dispersed in ethanol) were mounted on carbon-coated copper grids, double stained with freshly prepared uranyl acetate for 20 min, then with lead citrate for 2–5 min prior to imaging by TEM. The particle-size distribution of the synthesized CQDs was determined from TEM micrographs using ImageJ software (National Institutes of Health, Bethesda, MD, USA, version 1.53). Individual CQD particles with clearly identifiable boundaries were measured manually using the scale bar provided in the corresponding TEM images. Measurements were collected from representative TEM micrographs obtained from different regions of the sample. The measured particle diameters were used to construct a particle-size histogram, and the distribution was fitted using a Gaussian function. The mean particle diameter obtained from the distribution was used to represent the average CQD size.

#### 4.4.5. Scanning Electron Microscopy (SEM)

The microstructure and surface morphology of gelatin and G/CQD nanocomposite hydrogels were examined using SEM (JEOL 5300 JSM, Tokyo, Japan) operated at 15 keV. Prior to imaging, the samples were frozen under liquid nitrogen, mounted, and sputter-coated with gold to a thickness of 400 Å for 210 s using a sputter coating unit (JFC 1100 E). Pore-size measurements were performed using ImageJ software by calibrating each SEM image according to its corresponding scale bar. Representative pores with clearly defined boundaries were measured, and the resulting values were used to calculate the average pore size and standard deviation.

#### 4.4.6. UV/Visible and Photoluminescence Spectroscopy

UV/Visible absorption spectra were recorded in the 200–800 nm range using a Lambda 35 UV/Vis spectrophotometer (PerkinElmer, Waltham, MA, USA). Additionally, room-temperature photoluminescence (PL) spectra were obtained with excitation wavelengths ranging from 300 to 34 nm using a PerkinElmer LS 55 fluorescence spectrometer (Waltham, MA, USA).

#### 4.4.7. Zeta Potential Measurements

The zeta potential (ζ) of the prepared CQDs and G/CQD nanocomposite hydrogels was measured using laser Doppler velocimetry with a Zetasizer (Malvern, UK). The samples were diluted in deionized water and analyzed at 25 °C using a standard 1.0 cm quartz cell. The measurements were performed to evaluate the surface charge and colloidal stability of the prepared systems.

### 4.5. Mechanical Tests

The compressive properties of the G/CQD nanocomposite hydrogels were evaluated using a DMA 7e dynamic mechanical analyzer (PerkinElmer, Waltham, MA, USA) equipped with Pyris software (Version 10.1). Non-lyophilized cylindrical samples measuring 15 mm in diameter and 10 mm in height were subjected to unconfined uniaxial compression at room temperature. A stress rate of 500 mN/min was applied across a range from 100 to 6000 mN at a frequency of 1 Hz. To satisfy statistical replication and ensure reproducibility, a minimum of five independent specimens were tested to failure for each hydrogel formulation to determine the ultimate compressive strength. The compressive modulus was calculated from the average slope of the initial linear region corresponding to 10 to 20% strain of the stress–strain curve [65]. These tests were conducted to evaluate the structural integrity of the hydrogels in their fully hydrated state, thereby simulating their performance as porous templates for cell infiltration and mineral deposition. Prior to testing, the hydrated cylindrical specimens were carefully removed from the hydration medium, and excess surface water was gently blotted without applying mechanical pressure. Compression measurements were performed immediately to minimize any changes in sample hydration during handling.

### 4.6. Swelling Study

The swelling behavior of the G/CQD nanocomposite hydrogels was evaluated by incubating dried samples in distilled water at 25 °C. At predetermined time intervals, the hydrogels were removed from the medium, gently blotted with filter paper to eliminate excess surface water, and weighed. The degree of swelling (DS) was calculated via Equation (1) [66]:

(1) $$DS\left(\%\right)=\;\frac{(Ww-Wd)\;}{Wd}\;\times \;100$$

where W_d_ and W_w_ represent the dry and wet weights of the samples, respectively.

### 4.7. In Vitro Degradation Test

The degradation behavior of the hydrogels was evaluated in vitro by immersion in PBS, (pH 7.4) at 37 °C. Pre-weighed dry samples (Wi) were placed in sterile glass tubes containing 5 mL of PBS and incubated in a shaker at 50 rpm for 14 days. At designated intervals (1, 5, 7, and 14 days), samples were retrieved, rinsed with deionized water, and dried at 40 °C until a constant mass was achieved. The final dry weight (Wi) was recorded, and the degradation percentage was determined according to Equation (2) [31]:

(2) $$Degradation\;(\%)\;=\;\frac{\left(Wi-Wd\right)}{Wi}\;\times \;100$$

### 4.8. In Vitro Cytocompatibility and Cytotoxicity Assessment

The biological safety of the G/CQD scaffolds was validated through a cytotoxicity screening using Human Skin Fibroblasts (HSFs) obtained from Nawah Scientific, Inc. (Mokatam, Cairo, Egypt). This assessment confirmed that both the oatmeal-derived nanostructures and the DHT-cross-linked matrix demonstrate favorable preliminary cytocompatibility and support cellular viability. This cell line serves as a highly sensitive and established model for assessing cell–material interactions and ensuring the compatibility of the synthesized nanocomposites. To evaluate the potential for residual leaching in a physiological environment, extracts were prepared by incubating the sterilized scaffolds in DMEM for 24 h at 37 °C. HSF cells were seeded into 96-well plates at a density of 3.0 × 105 cells/mL (100 µL/well) and incubated for 24 h at 37 °C in a 5% CO2 atmosphere to facilitate attachment. The culture medium was then replaced with scaffold extracts and incubated for an additional 24 h. Following incubation, the cell monolayers were rinsed three times with sterile 1× PBS. To each well, 20 µL of MTT solution (5 mg/mL) was added, followed by a 4 h incubation at 24 °C. The resulting formazan crystals were dissolved in 200 µL of acidified isopropanol. The absorbance (Abs) was measured at 540 nm using a multi-well plate reader (BMG LABTECH^®^ FLUOstar Omega, Bensheim, Germany). The percentage of cell viability was calculated relative to untreated control cells using Equation (3) [67]:

(3) $$Cell\;viability\left(\%\right)=\;\;\frac{{Abs}_{\;Sample}}{{Abs\;}_{Control}}\;\;\times \;100$$

### 4.9. In Vitro Bioactivity and Mineralization Potential

The capacity of the G/CQD scaffolds to facilitate the nucleation and growth of bone-like apatite was evaluated through immersion in SBF. This study focused on the physicochemical bioactivity of the nanocomposites, specifically, their ability to function as mineral-inducing templates. Following established protocols [20], scaffold samples were immersed in SBF and incubated at 37 °C for a duration of 14 days.

After the incubation period, the samples were retrieved, rinsed twice with deionized water and twice with ethanol to stop any further precipitation, and dried until a constant mass was achieved. The formation and crystallinity of the induced hydroxyapatite (HA) phase on the scaffold surfaces were characterized using XRD. The resulting diffraction patterns were indexed according to the Joint Committee on Powder Diffraction Standards (JCPDS) reference for HA (PDF No. 74-0566) [68]. Furthermore, the surface morphology and the microstructural evolution of the mineralized layers were examined using SEM to confirm the development of a biomimetic inorganic interface.

### 4.10. Statistical Analysis

All quantitative data are presented as mean ± standard deviation (SD). The number of independent specimens/experiments used for each analysis is indicated in the corresponding figure captions or experimental description. Statistical comparisons were performed using one-way analysis of variance (ANOVA), followed by Tukey’s post hoc multiple-comparison test. Statistical significance was considered at *p* < 0.05. Prior to ANOVA, the data were evaluated for distributional assumptions and homogeneity of variance using appropriate statistical tests.

## Data Availability

All data supporting the results of this article are included in the article and its Supplementary Materials.

## Supplementary Materials

The following supporting information can be downloaded at https://www.mdpi.com/article/10.3390/gels12090757/s1, Figure S1: Zeta potential distribution of oat-derived carbon quantum dots (CQDs) in aqueous dispersion. The peak at −39.9 mV confirms high electrostatic stability and a robust surface functionalization, preventing nanoparticle aggregation.; Figure S2: XRD patterns of pristine gelatin (G) and G/CQD nanocomposite hydrogels with varying CQD loadings (5%, 10%, 15%, and 20%).
