# Supplementary material for: Green Synthesis of Oat-Derived Carbon Quantum Dot/Gelatin Hydrogel Scaffolds: Enhanced Structural Stability and Bioactivity for Potential Bone Repair
Source: Gels. 2026 Aug 24;12(9):757. doi: 10.3390/gels12090757 (PMC13606743; doi:10.3390/gels12090757)
Supplement: Supplementary file 1 [file gels-12-00757-s001.zip › gels-4482120-supplementary.pdf]

Green Synthesis of Oat-Derived Carbon Quantum Dot/Gelatin Hydrogel Scaffolds: Enhanced Structural Stability and Bioactivity for Potential Bone Repair

Supplementary Information

Section S1. Colloidal stability and surface charge analysis of CQDs

			Mean (mV)	Area (%)	St Dev (mV)
Zeta Potential (mV):	-39.9	Peak 1:	-39.9	100.0	9.75
Zeta Deviation (mV):	9.75	Peak 2:	0.00	0.0	0.00
Conductivity (mS/cm):<br>Result quality:	0.145<br>Good	Peak 3:	0.00	0.0	0.00

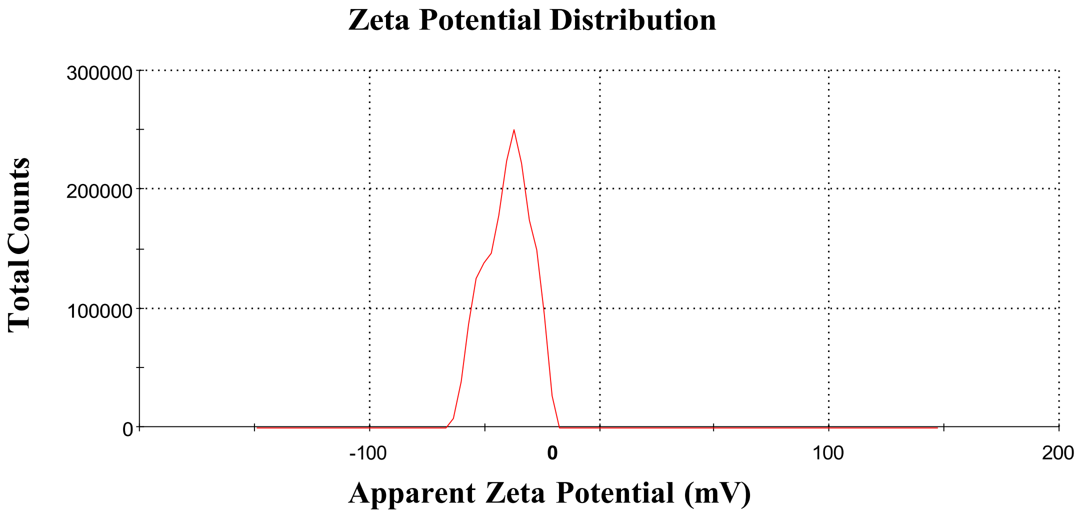

Figure S1 Zeta potential distribution of oat-derived carbon quantum dots (CQDs) in aqueous dispersion. The peak at -39.9 mV confirms high electrostatic stability and a robust surface functionalization, preventing nanoparticle aggregation.

The surface charge of the synthesized CQDs was investigated to evaluate their stability and suitability for hydrogel integration. As shown in Fig. S1, the CQDs exhibit a sharp peak at -39.9 mV. This strong negative charge arises from the intrinsic oxygen-containing groups (e.g., carboxyl and hydroxyl moieties) formed during the hydrothermal carbonization of oatmeal. This self-functionalization eliminates the need for additional chemical surface modification, ensuring that the nanostructures remain additive-free while maintaining superior colloidal stability in aqueous media compared to traditional carbon precursors.

Section S2. XRD of gelatin and G/CQD nanocomposite hydrogels

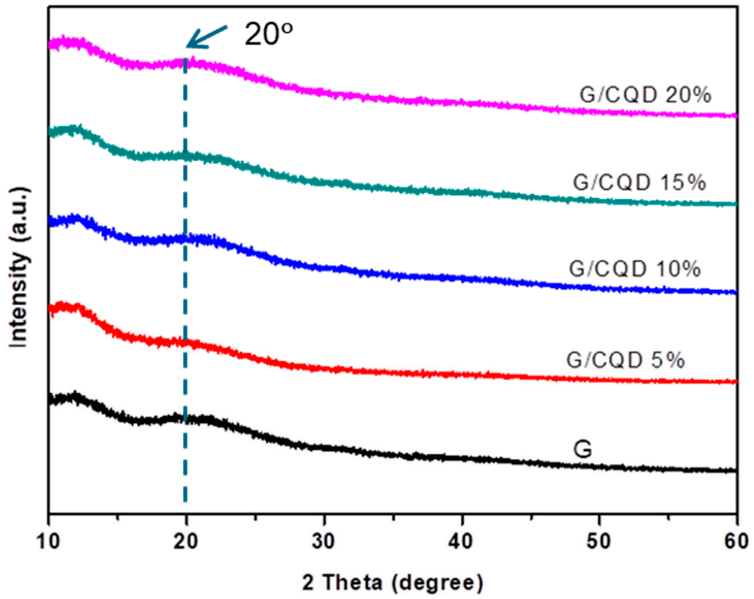

Figure S2. XRD patterns of pristine gelatin (G) and G/CQD nanocomposite hydrogels with varying CQD loadings (5%, 10%, 15%, and 20%).
